# Transcriptome responses to heat stress in hypothalamus of a meat-type chicken

**DOI:** 10.1186/s40104-015-0003-6

**Published:** 2015-02-17

**Authors:** Hongyan Sun, Runshen Jiang, Shengyou Xu, Zebin Zhang, Guiyun Xu, Jiangxia Zheng, Lujiang Qu

**Affiliations:** Department of Animal Genetics and Breeding, National Engineering Laboratory for Animal Breeding, College of Animal Science and Technology, China Agricultural University, Beijing, 100193 China; College of Animal Science and Technology, Anhui Agricultural University, Hefei, 230036 China

**Keywords:** Chicken, Heat shock protein, Heat stress, Hypothalamus, Microarray, Transcription

## Abstract

**Background:**

Heat stress has resulted in great losses in poultry production. To address this issue, we systematically analyzed chicken hypothalamus transcriptome responses to thermal stress using a 44 k chicken Agilent microarray,

**Methods:**

Hypothalamus samples were collected from a control group reared at 25°C, a heat-stress group treated at 34°C for 24 h, and a temperature-recovery group reared at 25°C for 24 h following a heat-stress treatment. We compared the expression profiles between each pair of the three groups using microarray data.

**Results:**

A total of 1,967 probe sets were found to be differentially expressed in the three comparisons with *P* < 0.05 and a fold change (FC) higher than 1.5, and the genes were mainly involved in self-regulation and compensation required to maintain homeostasis. Consistent expression results were found for 11 selected genes by quantitative real-time PCR. Thirty-eight interesting differential expression genes were found from GO term annotation and those genes were related to meat quality, growth, and crucial enzymes. Using these genes for genetic network analysis, we obtained three genetic networks. Moreover, the transcripts of heat-shock protein, including Hsp 40 and Hsp 90, were significantly altered in response to thermal stress.

**Conclusions:**

This study provides a broader understanding of molecular mechanisms underlying stress response in chickens and discovery of novel genes that are regulated in a specific thermal-stress manner.

**Electronic supplementary material:**

The online version of this article (doi:10.1186/s40104-015-0003-6) contains supplementary material, which is available to authorized users.

## Introduction

With increasing importance of global warming issues, heat stress, also known as hyperthermia, could play a vital factor in affecting animal performance. Although chickens can use a variety of physiological mechanisms to regulate their body temperatures, heat stress could adversely affect chickens, especially broilers, in matters such as high mortality and consistent decrease in egg weight, shell thickness, rate of egg production, growth rate, meat quality, body weight [1,2], and immune capability as well as blood biochemical indicators [3,4]. St. Pierre et al. [5], reported that heat stress caused economic losses of between $1.69 billion and $2.36 billion per year in livestock industries in United States, with $51.8 million in the broiler sector alone.

During the past few decades, researchers have paid increased attention to physiological, biochemical, and immune capability changes of heat-stressed chickens. Deyhim and Teeter [6] found physiological responses to heat stress involve changes in respiration rate and blood pH, plasma concentration of ions, cardiovascular functions and hormonal effects. Heat stress also increased glucose and circulatory cortisol levels and decreased blood protein level. Khajavi et al. [7], reported that occurrence of CD4^+^ and CD8^+^ lymphocytes and antibody production decreased under heat stress.

Recently, transcriptome comparison has become a popular methodology for studying heat stress because it can elucidate the heat stress mechanism based on genetic level. Li et al. [8], using broiler breast tissue, investigated 110 differentially expressed genes during chronic cyclic heat stress, including 4 new genes that were related to heat stress. Wang et al. [9], reported 169 up-regulated and 140 down-regulated genes responded to acute heat stress; most of the differential expression genes were related to transport, signal, and metabolism.

Previous transcriptome research studies have chosen liver, breast, and testis as materials [8-10] to decipher heat stress, but no one has used the hypothalamus as a subject for studying the mechanism of heat stress and genetic networks. The hypothalamus, linking the nervous system to the endocrine system via the pituitary gland, plays the role of a central regulator in temperature regulation in poultry. It is well known the preoptic area (POA) and anterior hypothalamic area (AH) of the hypothalamus is the thermoregulatory center [11] and that hypothalamic areas such as the paraventricular hypothalamic nucleus (PVN), supraoptic nucleus (SO) [12-14], and lateral hypothalamic (LH) are activated under heat stress [15,16].

In addition, although RNA sequencing is currently a popular technology for studying transcriptome, it is very costly and results in less mature analysis than a microarray. The objective of this study is thus to use a microarray to detect the versatile gene expression profile in the hypothalamus, to determine the genes associated with heat stress, and to gain insight into the potential mechanisms underlying chickens’ response to heat stress. Characterizing the hypothalamus transcriptome of heat-stressed broilers will help clarify the effects of heat stress on broiler nerve centre and signal transduction. This information will also provide a platform for future investigation into genetic networks relevant to commercial broilers’ response to heat stress.

## Materials and methods

### Experimental animals and sampling

All experimental protocols were approved by the Committee for the Care and Use of Experimental Animal at Anhui Agricultural University. The purpose of this study was to find how broiler hypothalamus responds to heat stress in influencng growth rate and meat quality at the genetic level. Moreover, considering the particular economic value of hens, our priority was to use hen broilers as research subjects. Because broilers don’t have sweat glands, it is difficult for them to shed heat in hot weather (30-35°C). At a temperature of 34°C with 10-50% relative humidity, 50-80% of the heat loss was by evaporative heat shed through panting (http://www.heatstress.info/HeatStressExplained/BodyHeatregulationinPoultry.aspx). In this study, we used a full-sib and half-sib chicken population to reduce the impact of genetic background variation. Thus, twelve 56-day-old full-sib and half-sib commercial female broilers were randomly divided into three groups, including a control group, a heat-treated group, and a temperature-recovery group with four replicates in each group. All the birds were initially reared in an artificial climate chamber (relative humidity of 40-60%) with free access to food and water at 25°C. At the age of 56 days, both the heat-treated (HS) group and the temperature-recovery (TR) group were subjected to a temperature of 34°C for 24 h. After heat stress, hypothalamuses of four HS hens were dissected from their brains. The other chickens as a control group (CL) were further kept at a normal temperature of 25°C for 24 h. After exposure to heat, the hypothalamuses of CL and TR groups were collected. The hypothalamus collection procedure was as follows: (1) After decapitation, carefully remove the brain; then bisect it by making a section perpendicular to the cortical surface at the caudal end of the hemisphere. (2) Before identifying the hypothalamus, carefully remove the meninges and then, using tweezers, carefully pull the meninges layers away from the brain. (3) When the hypothalamus has been found, carefully remove the whole specimen. All the tissues were preserved overnight in RNAfixer (BioTeke Co. Ltd, Beijing, China) at 4°C and transferred to −80°C until RNA isolation. The hypothalamuses from the three groups were then subjected to gene expression analysis.

### Total RNA isolation, labeling, and microarray hybridization

Total RNA was isolated from the hypothalamus using TRIZOL Reagent according to manufacturer’s instructions (Life Technologies, CA, US). RNA quantity and purity of each sample were detected using a NanoDrop ND-1000 spectrophotometer (Thermo Fisher Scientific Inc, Delaware, US). The RNA integrity of each sample was determined using an Agilent 2100 Bioanalyzer. The processed RNA had a 28S/18S > 1.5 and the RNA Integrity Numbers (RINs) for the samples were all higher than 7.6, indicating that the quality of the samples was suitable for microarray analysis. Four biological replicates were used in each group and cyanine 3-labeled nucleotide was conducted to prevent dye bias during sample labeling. A 2 μg isolated total RNA from each tissue sample was reverse-transcribed into cDNA, then into cRNA incorporating cyanine 3-labelled nucleotide. The labeled RNA was purified using QIAGEN RNeasy® Mini Kit (Qiagen, CA, US). A total of 875 ng Cy3 labeled cRNA was hybridized to 44 K chicken Agilent arrays (ID: 026441). Equal amounts of cRNA were hybridized using a Gene Expression Hybridization Kit (5188–5242, Agilent, USA). Arrays were performed at 60°C for 17 h in Agilent microarray hybridization chambers. The hybridized slides were washed using a Gene Expression Wash Buffer Kit (Agilent technologies, Santa Clara, CA, US) before stabilization and drying solutions were applied (Agilent Technologies, CA, US). Arrays were scanned using an Agilent Microarray Scanner (Agilent Technologies, Santa Clara, CA, US) with a scan resolution of 5 μmol/L, PMT 100%, 10%. The data were then compiled with Feature Extraction software 10.7 (Agilent Technologies, Santa Clara, CA, US).

### Microarray data analysis

Array normalization and error detection analysis was carried out using a Quantile algorithm supplied with Gene Spring Software 11.0 (Agilent Technologies, CA, US). The “filter on flags feature” with software algorithms determined whether spots were “present”, “marginal”, or “absent” and values representing poor intensity and low dependability were removed from the raw data. The “absent” spots represented signals not positive, significant or above background level. A “present” spot was one for which the output was uniform, saturated, and significantly above background level. Spots were deemed “marginal” when the signals satisfied the main requirements but were outliers relative to the typical values for the other genes. We used only the items categorized as “present” and “marginal” for further analysis and compared the differential expression transcripts between each pair of the three groups. i.e., HS compared to CL (HS vs. CL), HS compared to TR (HS vs. TR), or TR compared to CL (TR vs. CL).

Principal-component analysis (PCA) and heat-map analysis were performed by SAS software from SHANGHAI BIOTECHNOLOGY CORPORATION eBioService (http://www.ebioservice.com). The differentially-expressed (DE) genes in each comparison were selected using SAS online software (http://www.ebioservice.com) and a local R package. With respect to the number of expression genes in the hypothalamus, we set the cutoff as *P* < 0.05, FDR < 0.15 and fold change (FC) > 1.5 to obtain more DE genes. Although this FDR cutoff is much higher than the usual FDR < 0.05, there are many good published papers using a variety of different FDR cutoff values according to reported experiments [17,18]. The significant DE gene clusters from heat-map results were used to do pathway analysis using Ingenuity Pathway Analysis (IPA) (Ingenuity® Systems, www.ingenuity.com). IPA is a very useful online tool for analyzing biological and molecular networks; it transforms gene network into relevant pathways using the genes’ functional annotation and known molecular interactions [19-21].

### Quantitative real time PCR (qRT-PCR)

Eleven genes were quantitatively determined using real-time PCR with the same 12 samples tested by the microarray to confirm gene-expression patterns observed using the microarray. PCR-specific primers for target genes were designed with Primer Express software (Version 3.0, Applied Biosystems, US) and were synthesized by Shanghai Sangong Biological Engineering Technology & Services Co, Ltd. (Shanghai, China). All the primers were sequenced and worked well. β-actin was used as the internal control for the target genes. Real-time PCR primer information is shown in Table 1. The reverse transcription reaction system was conducted in a total volume of 25 μL, 1 μg of total RNA, 10 mmol/L oligo T primer and murine leukemia virus (MLV) reverse transcriptase (Promega Biotech Co., Ltd, Beijing, China). The PCR system was quantified using the ABI 7300 system (Applied Biosystems, US) in a final volume of 15 μL with 1 μL of cDNA, 200 nmol/L of each primer and a 1 × PCR mix (Power SYBR® Green PCR Master Mix, Applied Biosystems, US). The optimum thermal cycles were performed as follows: denaturation at 95°C for 10 min, then 40 cycles of 95°C for 15 s, 60°C for 1 min, 95°C for 15 s, 60°C for 30 s, and 95°C for 15 s. Every sample was supplied in duplicate and the average critical threshold cycle (Ct) was used for calculating the relative quantification by fold-change and statistical significance. At the same time, to ensure that a single PCR product was amplified in each reaction, dissociation curve analysis at the end of amplification and gel electrophoresis was performed after the real-time PCR. The resulting data for each gene were calculated by the expression 2^-∆∆∆Ct^, where the -∆∆Ct is the sum of :[Ct _gene_ – Ct_β-actin_] (treatment) – [Ct _gene_ - Ct_β-actin_] (control) for the relative gene expression level among the three comparison groups. All analyses were subjected to a Student’s t-test to confirm statistically-significant gene expression.

**Table 1:** **Primer sequences for qRT-PCRs**

**Gene symbol**	**Accession no.**	**Forward (5′-3′)**	**Reverse (5′-3′)**	**Product size, bp**
DNAJC13	XM_418787	TGAAAGGAGCAGGTTTGGTGAT	GTGTGCAAGTGACGAGGTAAGG	115
HSPCB	NM_206959	CAGGCGCAGACATCTCCAT	TGACGACTCCCAGGCATACTG	125
HMOX1	NM_205344	TCCCTCCACGAGTTCAAGCT	CGGAAAATAAACAGGAGCATAGACA	114
TH	NM_204805	GAGAGCCATGCTGAACCTGTT	GGGCTTTCGGCTGAGTCTT	130
EDN3	AB235921	TTTCGGTGCTCTTGTTTGGAT	CGGTCCTTCTCTGTTGTTTTCAC	110
GCH1	NM_205223	CAGGAACGCCTTACCAAACAA	CTGGTTGCCGTTTTACTGTTCA	140
GPR23	CR353562	TCAGATCGGGACAAACAAAGAGA	CCGCACGAGAGCATACAAGA	115
SDC1	XM_419972	TGTAGTGACAGAGGAGCCAGTTG	TGAAGCACCGAAGGGAAGTG	135
PDK4	CR387492	AGTTCCATCAGAAAAGCCCAGAT	TCCTTGTGCCATTGTAGGGACTA	110
ITGA8	NM_205288	TAGCCGTGGCAGAACCTTACA	CCATAGCCGGGACCTGATTT	140
SFRP1	NM_204553	CCAGTTCCCACAGGATTATGTCT	CCTCCGACTTCATCTCGTTGTC	116
β-actin	NM_205518	GAGAAATTGTGCGTGACATCA	CCTGAACCTCTCATTGCCA	132

## Results

### Effects of heat stress on gene expression in different comparison groups

**Figure 1:**
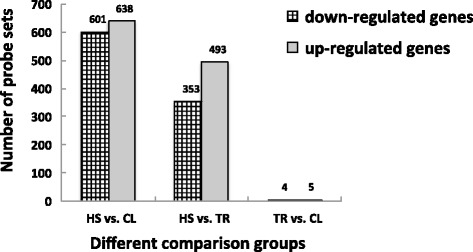
**The number of differentially expressed genes in three different comparison groups.** Note: FDR < 0.05 and fold change ≥ 1.5; HS: heat stress group; CL: control group; TR: temperature recovery group.

**Figure 2:**
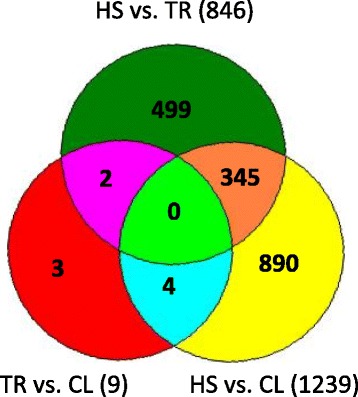
**Differentially expressed genes unique and shared between different contrast groups.** Note: FDR < 0.05 and fold change ≥ 1.5; HS: heat stress group; CL: control group; TR: temperature recovery group.

A comparative analysis was performed by comparing gene expression profiling between each pair of the three groups CL, HS, and TR. Generally, more DE genes were present in the comparison of HS vs. CL and HS vs. TR compared to that of TR vs. CL. In summary, there were 1,239, 846, and 9 probe sets DE in HS vs. CL, HS vs. TR, and TR vs. CL, respectively. These results showed that the TR group is more similar to the CL group. Moreover, 638 of 1,239 probe sets, 493 of 846 probe sets and 5 of 9 probe sets are up-regulated in HS vs. CL, HS vs. TR, and TR vs. CL, respectively (Figure 1). The FC range was 1.5 to 25 in HS vs. CL, 1.5 to 36 in HS vs. TR, and 1.5 to 16 in TR vs. CL. In addition, the pattern of the total 1,743 differential expression probe sets is shown in Figure 2. There were 890, 499, and 3 probe sets uniquely DE in HS vs. CL, HS vs. TR, and TR vs. CL, respectively. Contrasts HS vs. CL and HS vs. TR shared 345 DE genes (Figure 2). All the microarray data have been deposited in the GEO repository (GSE37400, NCBI tracking system #16562670).

### Heat map and Principal component analysis

**Figure 3:**
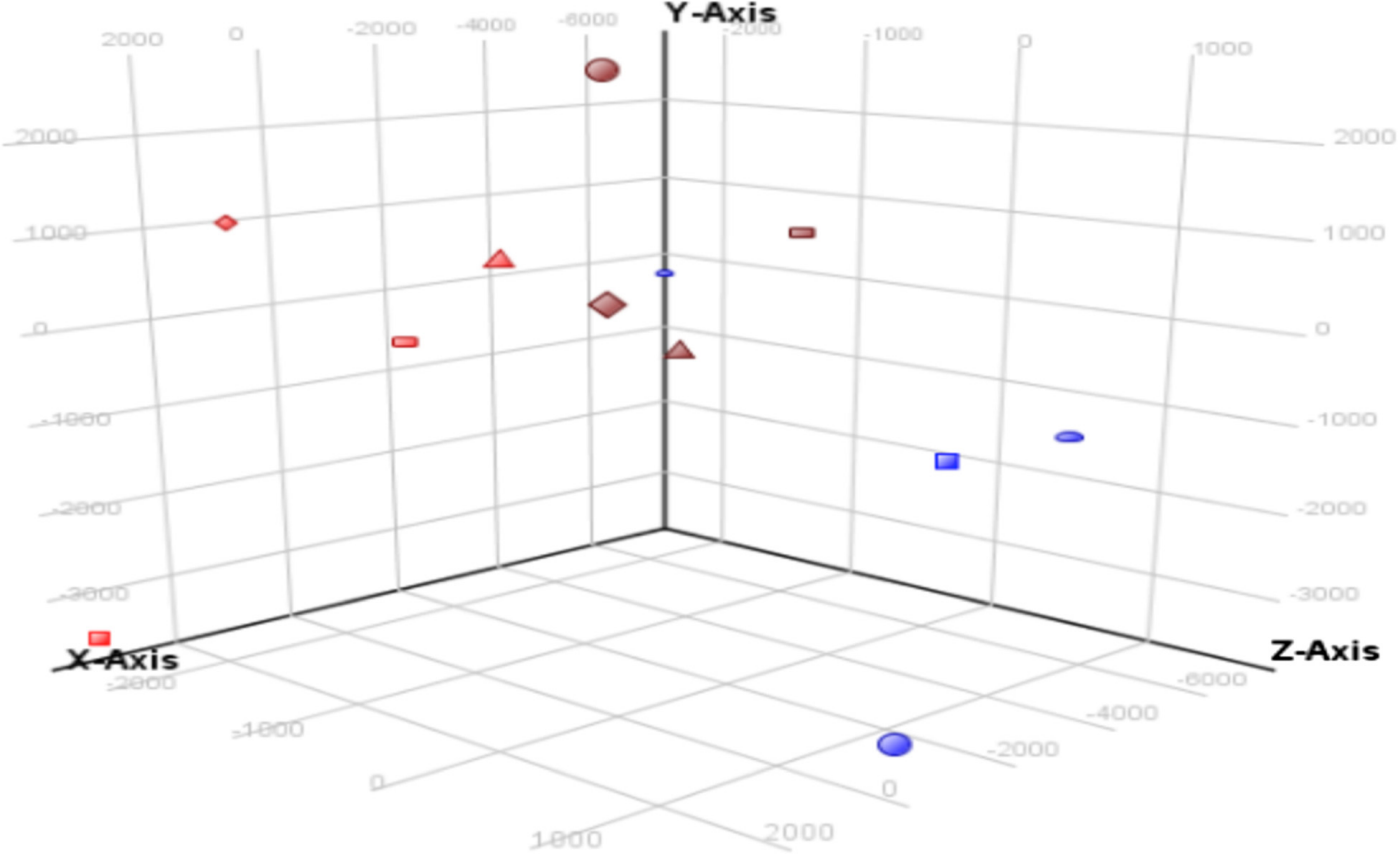
**Principal component analysis.** Note: red color is control group; blue color is heat stress group; brown color is temperature recovery group.

**Figure 4:**
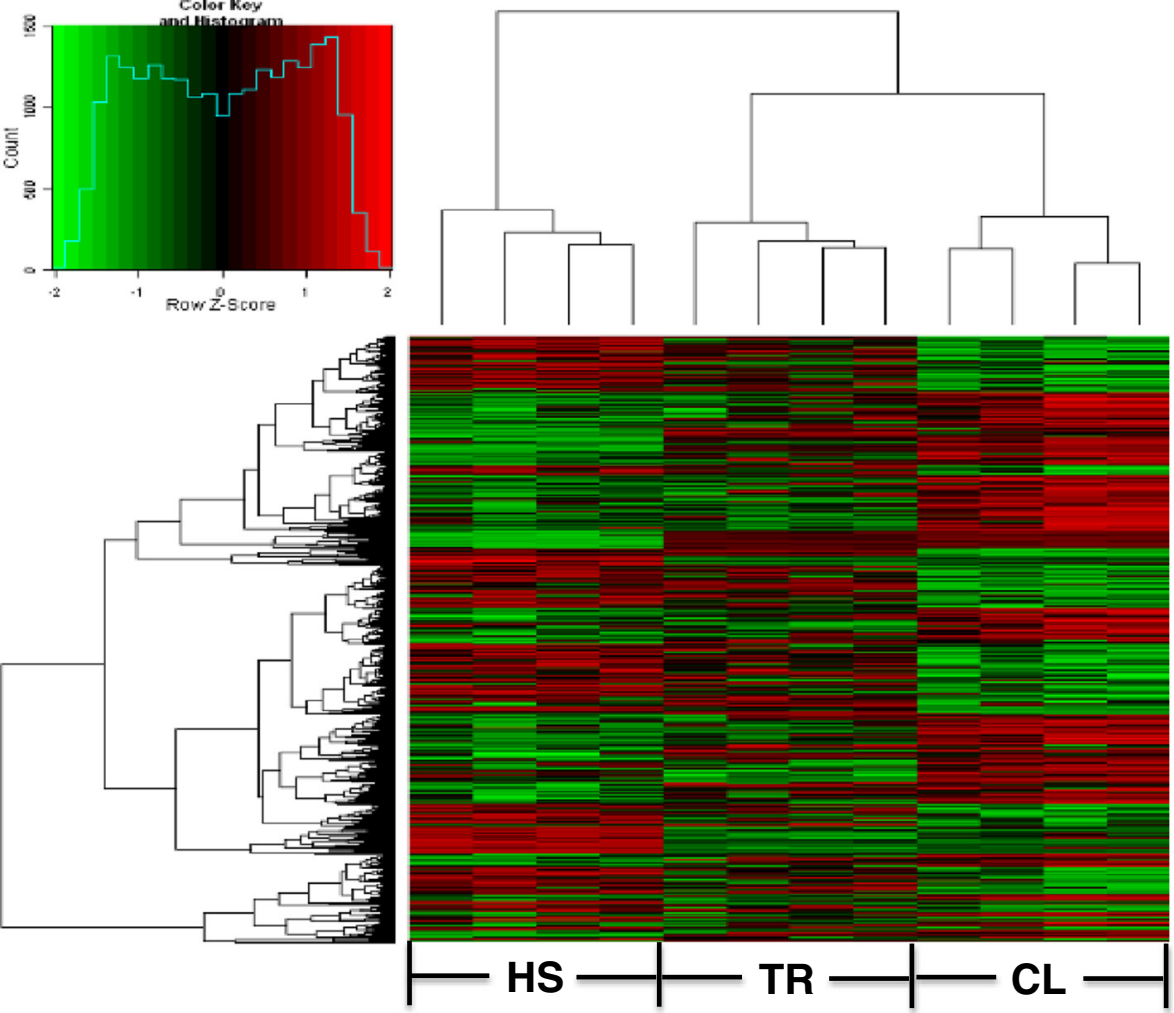
**Heat map.** The X-axis is sample expression pattern in different treatment group. The first four samples are heat stress (HS) treatment. The middle four samples are temperature recovery (TR) treatment after heat stress. The last four samples are control group. The Y-axis is the gene clusters across HS, TR, and CL treatment.

Principal component analysis (PCA) was used to classify the broilers based on their gene expression patterns in one-way analysis of variance (ANOVA) with *P* < 0.05 and FC > 1.5 among three groups (12 samples). The HS group had a transcription profile different from those of the CL and TR groups, proving that heat stress reprograms gene expression in broiler hypothalamus and has a unique expression pattern. Using PCA to observe the clustering of samples, we found that gene expression of HS group showed the largest variation, with the four samples distributing over a wide range. One sample of HS treatment was located in TR treatment, which may be because that bird responded to heat stress very quickly and had its own method for protecting itself. The four CL samples were highly homogenous, while the four TR chickens exhibited proximal patterns distributed similarly to the CL samples (Figure 3). Moreover, a heat map was also developed to investigate the expression pattern and gene clusters (Figure 4). The heat map result is consistent with the PCA result in terms of sample expression pattern. The gene clusters were also subjected to further analysis similar to the gene network analysis (IPA).

### Functional categories of differentially expressed genes

**Figure 5:**
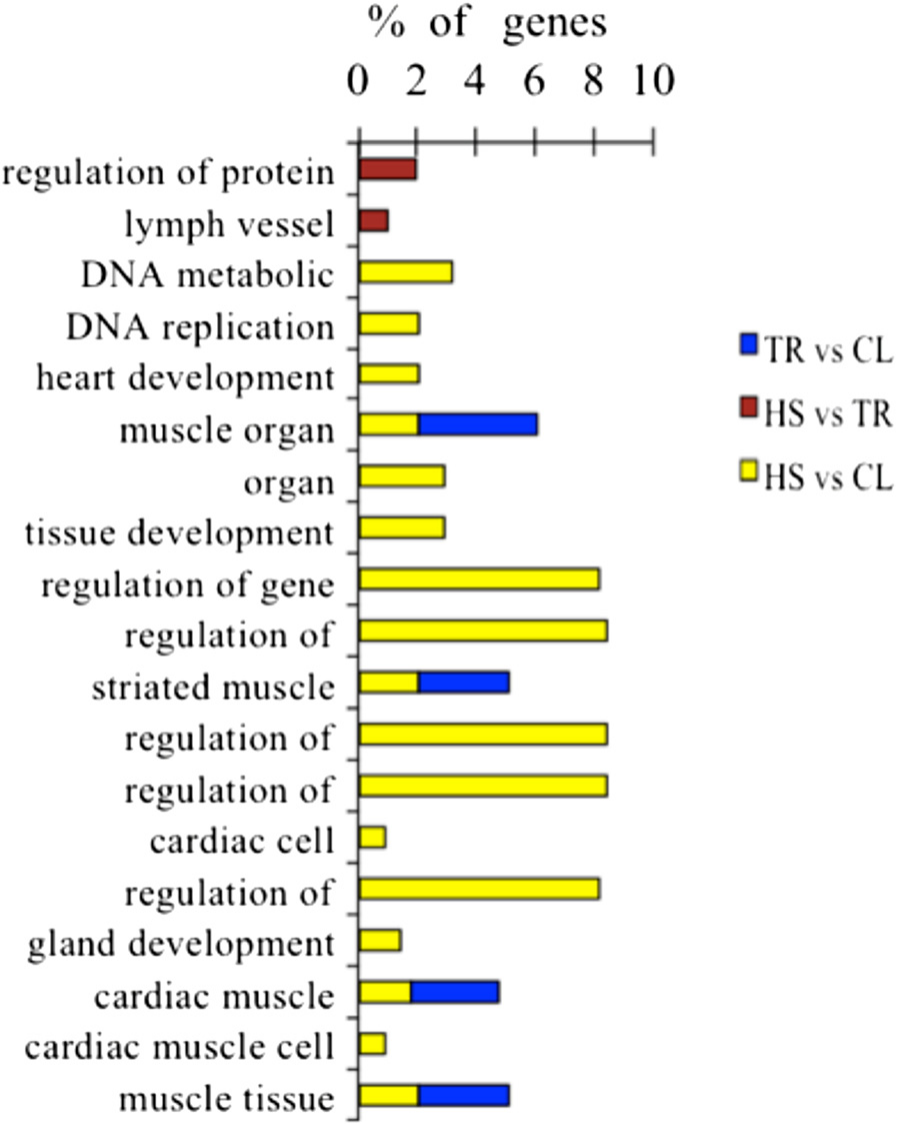
**Gene ontology (GO) annotation of down-regulated differentially expressed genes (P < 0.05).**

**Figure 6:**
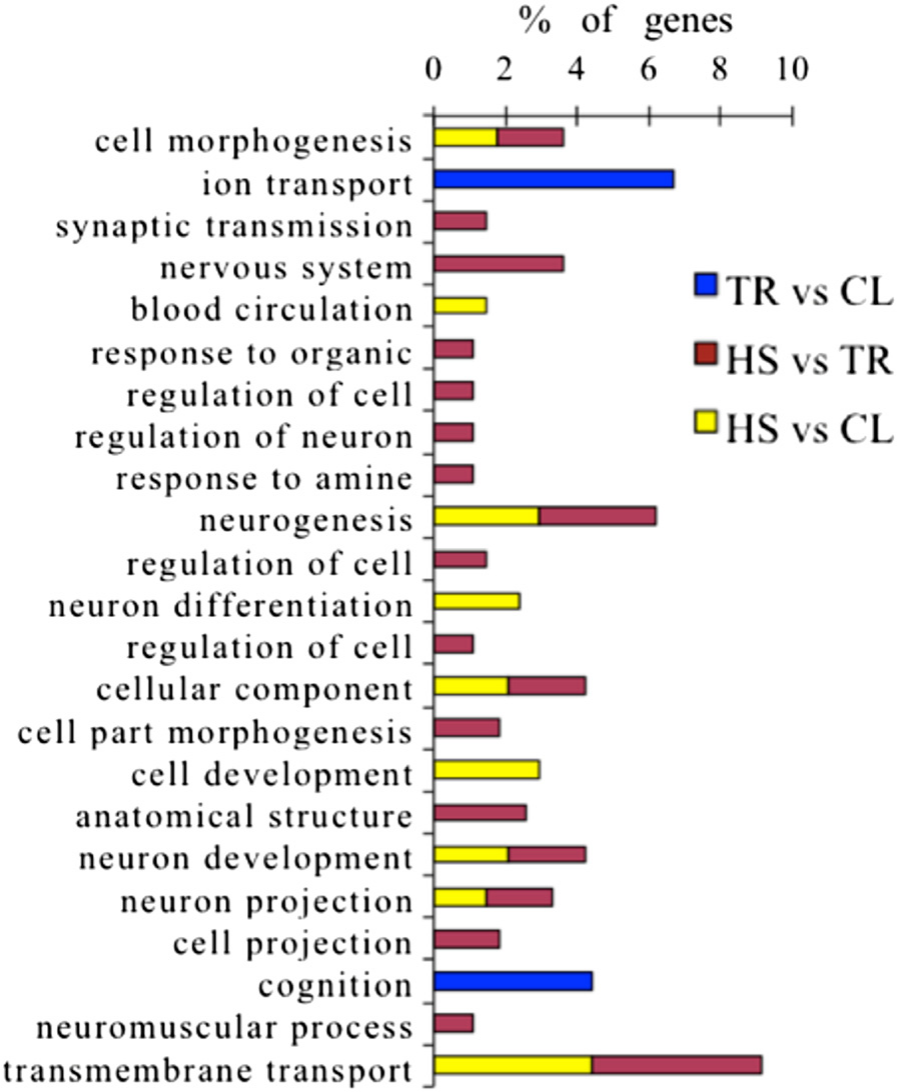
**Gene ontology (GO) annotation of up-regulated differentially expressed genes (P < 0.05).**

Gene ontology was used to evaluate the function of DE genes in three comparisons (Figures 5 and 6). All the DE genes in HS vs. CL, HS vs. TR, and TR vs. CL were performed by gene ontology (GO) terms through the DAVID platform. The biological process (BP) components were presented as functional clusters. The significantly-enriched GO categories were selected only when *P* < 0.05 at the fifth level. In summary, up-regulated genes in HS vs. CL and HS vs. TR were mainly enriched in regulation of cell morphogenesis involved in differentiation, neurogenesis, cellular component morphogenesis, neuron development, neuron projection morphogenesis, and transmembrane transport. GO items such as ion transport and cognition were associated only with down-regulation in TR vs. CL. The down-regulated genes between HS vs. CL and TR vs. CL showed muscle-organ development, striated muscle-tissue development, and cardiac muscle-tissue development; muscle-tissue development was enriched, indicating that muscle development is significantly inhibited during heat stress. In HS vs. CL, the up-regulated genes were mostly concentrated in regulation of gene expression, regulation of macromolecule biosynthetic process, regulation of nucleobase, nucleoside, nucleotide and nucleic acid metabolic processes, regulation of transcription, and DNA metabolic processes. The regulation of protein amino acid phosphorylation and lymph vessel development were only changed in HS vs. TR. Also, among the many functional GO annotation categories, a number of genes were involved that might relate to or affect meat quality, growth factor, and enzyme (Additional file 1: Table S1).

**Figure 7:**
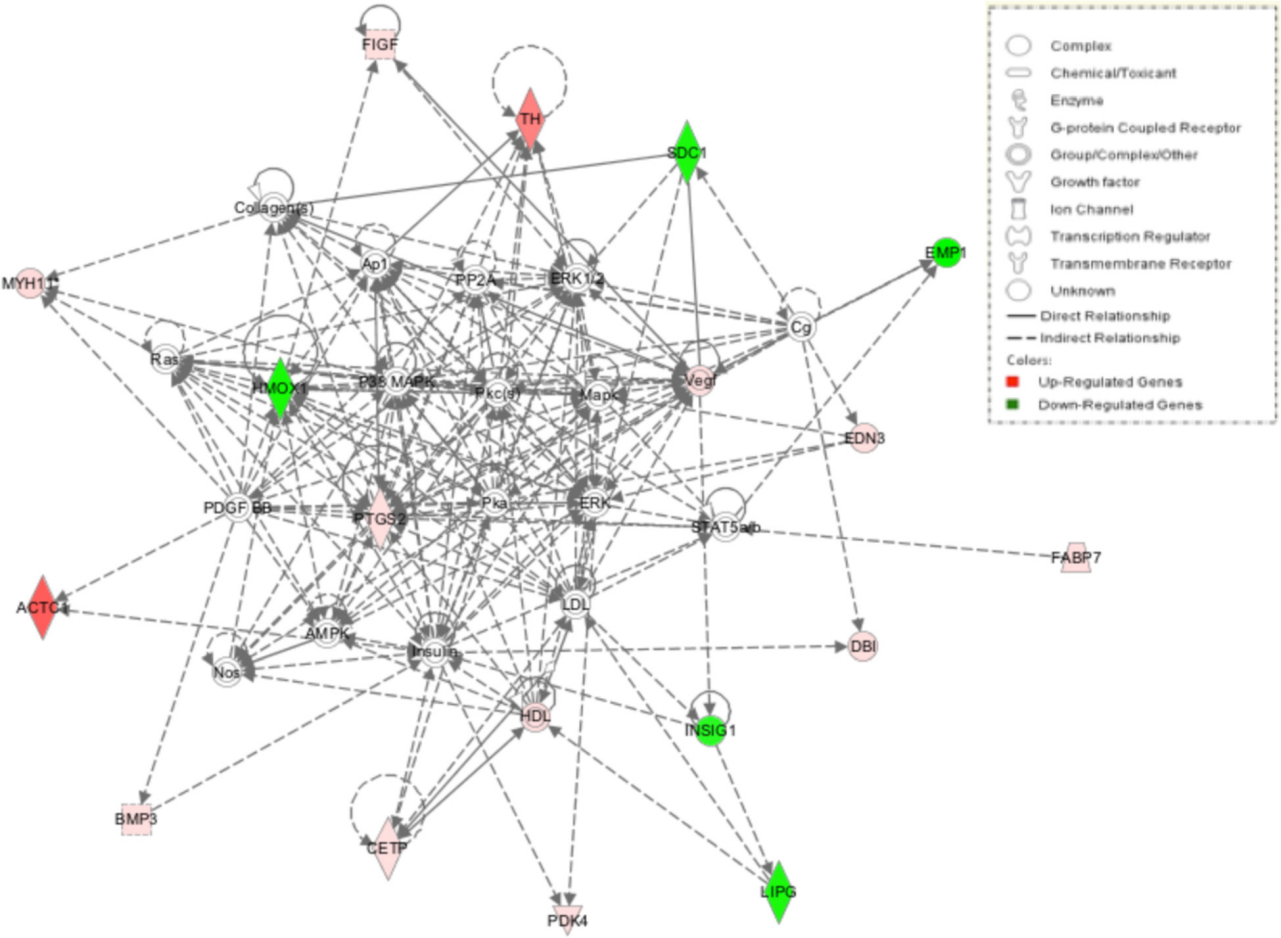
**Cardiovascular disease, lipid metabolism, molecular transport.** Pathway analysis of gene functions in broiler hypothalamus transcriptome in response to acute heat stress. Red color shows up-regulation and green color shows down-regulation (IPA). The intensity of green and red molecule colors indicates the degree of down or upregulation, respectively. White molecules are not differential expression, but are included to illustrate association with significantly up-regulated and down-regulated genes.

**Figure 8:**
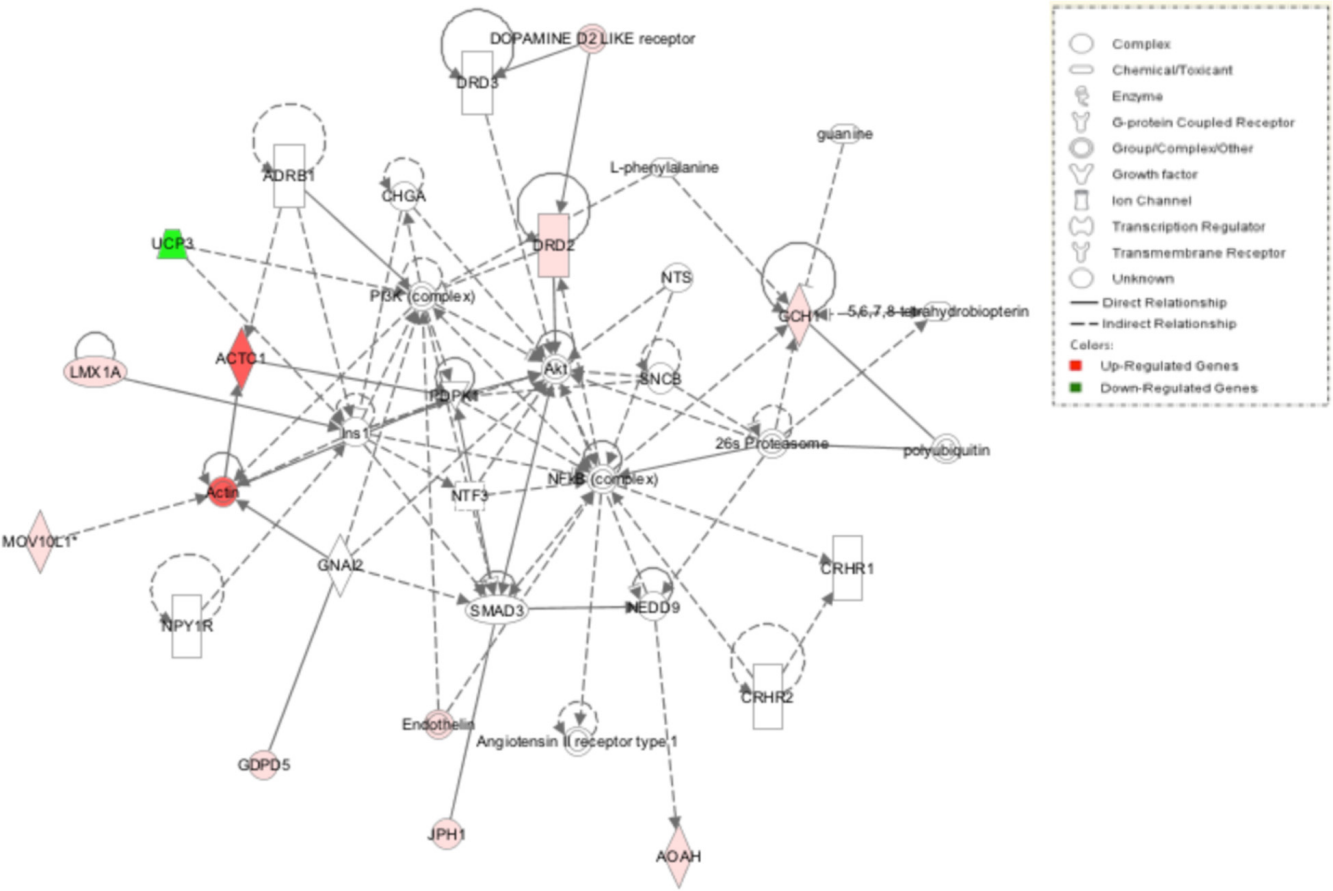
**Cell-cell signaling and interaction, molecular transport, small molecular biochemistry.** Pathway analysis of gene functions in broiler hypothalamus transcriptome in response to acute heat stress. Red color shows up-regulation and green color shows down-regulation (IPA). The intensity of green and red molecule colors indicates the degree of down or upregulation, respectively. White molecules are not differential expression, but are included to illustrate association with significantly up-regulated and down-regulated genes.

**Figure 9:**
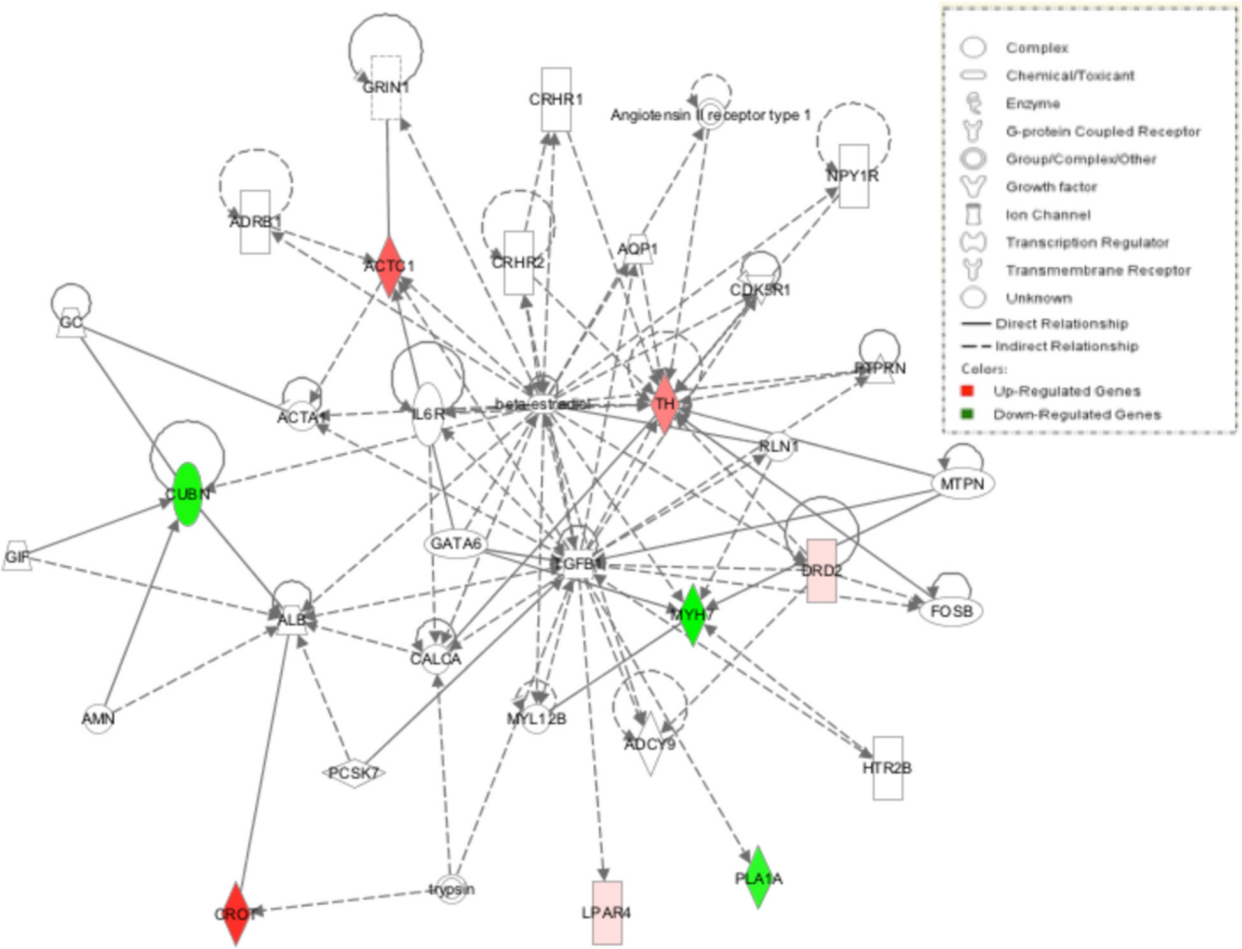
**Lipid metabolism, molecular transport, small molecule biochemistry.** Pathway analysis of gene functions in broiler hypothalamus transcriptome in response to acute heat stress. Red color shows up-regulation and green color shows down-regulation (IPA). The intensity of green and red molecule colors indicates the degree of down or upregulation, respectively. White molecules are not differential expression, but are included to illustrate association with significantly up-regulated and down-regulated genes.

Based on the fact that genes might be related to meat quality, growth factor, and enzyme from the GO annotation, a functional network of these DE genes and their interrelationships were analyzed using Ingenuity Pathway Analysis (IPA); three genetic networks (*P*-value = 0.0001) were created from those DE genes: “Cardiovascular disease, lipid metabolism, molecular transport” (*ACTC1*, *BMP3*, *CETP*, *DBI*, *FABP7*, *EMP1*, *FIGF*, *LIPG*, *MYH11*, *PDK4*, *PTGS2*, *SDC1*, and TH), “Cell-to-cell signalling and interaction, molecular transport, small molecule biochemistry” (*ACTC1*, *AOAH*, *DRD2*, *GCH1*, *GDPD5*, *JPH1*, *LMX1A*, *LOC430178*, and *UCP3*), and “Lipid metabolism, molecular transport, small molecule biochemistry” (*CROT*, *CUBN*, *DRD2*, *LPAR4*, *MYH7*, *PLA1A*, and *TH*) (Figures 7, 8 and 9).

### Validation of quantitative real time PCR results

We detected 11 DE genes using qRT-PCR to verify our microarray analysis. These eleven genes were distributed in three comparison groups. *DNAJC13* (HSP40), *HSPCB* (HSP90), *TH*, *GCH1*, *GPR23* were DE in both HS vs. CL and HS vs. TR. *ITGA8* transcripts significantly differed in both HS vs. TR and TR vs. CL. *HMOX1*, *EDN3*, *SDC1*, *PDK4* were uniquely changed in HS vs. CL and SFRP was altered in TR vs. CL. Among the 11 tested genes, two genes (*DNAJC13*, *HSPCB*) were included from the heat-shock protein family; four DE genes (*TH*, *HMOX1*, *GCH1*, *GPR23*) were related to crucial enzymes under heat stress; three DE genes (*SDC1*, *EDN3*, *SFRP*) were involved in growth, and the other two DE genes were randomly selected from our DE genes data set. The qRT-PCR results showed that the eleven selected genes presented the same expression pattern as those in the microarray although the fold change differed somewhat for the two methods. We thus have confidence in the reliability of the microarray results (Table 2).

**Table 2:** **Fold change of 11 selected genes in each comparison group by microarray and qRT-PCR**

**Gene symbol**	**HS vs CL fold-change**		**HS vs TR fold-change**		**TR vs CL fold-change**	
	**Microarray**	**qRT-PCR**	**Microarray**	**qRT-PCR**	**Microarray**	**qRT-PCR**
HSPCB	−1.61*	−1.94*	−1.53*	−1.52*	--	--
DNAJC13	1.90**	1.74**	1.95**	1.53*	--	--
TH	11.76*	12.30*	18.86*	12.70**	--	--
GCH1	2.64**	2.51*	2.42*	1.54*	--	--
HMOX1	−1.63*	−1.94*	--	--	--	−1.85*
EDN3	1.82*	2.12*	--	--	--	--
GPR23	1.59**	2.79*	--	--	--	--
SDC1	−1.61*	−1.55*	--	--	--	--
PDK4	2.48*	1.67**	--	--	--	--
ITGA8	--	--	−3.38**	−1.46*	−2.51*	−1.12
SFRP1	--	--	--	--	1.57**	1.55*

*Means significant difference between the treatments (*P <* 0.05). **means very significant difference between the treatments (*P <* 0.01). “--” means no significant difference between the treatments or the fold change is less than 1.5.

## Discussion

Much literature has shown that meat quality and growth can be influenced by environmental heat stress [22-25], but few studies explain this phenomenon at the gene level for broilers, especially with a focus on the hypothalamus. In this study we found 24 DE genes related to meat quality, 8 DE genes in the growth category, and 6 crucial DE enzyme genes associated with acute heat stress and based on heat-map gene clusters and GO term annotation. In addition, these meat quality and growth related DE genes, together with the crucial enzyme genes and other DE genes were subjected to genetic network analysis through IPA. We found 3 networks: Cardiovascular Disease, Lipid Metabolism, Molecular Transport; Cell-to-Cell Signaling and Interaction, Molecular Transport, Small Molecule Biochemistry; and Lipid Metabolism, Molecular Transport, Small Molecule Biochemistry.

### Gene network analysis

*Cardiovascular Disease, Lipid Metabolism, Molecular Transport*. The hub in the “Cardiovascular Disease, Lipid Metabolism, Molecular Transport” network is prostaglandin G/H synthase 2 precursor (*PTGS2*), also known as *COX2*, an enzyme that can catalyse the biosynthesis of prostaglandins (PGs) [26]. *PTGS2* directly interacts in this network with heme oxygenase (decycling) 1 (*HMOX1* or *HO1*), platelet-derived growth factor beta (*PDGFB*), insulin (Ins), vascular endothelial growth factor (*VEGF*), signal transducer and activator of transcription 5B (*STAT5B*), and so on. *HMOX1*, *PDGFB*, Ins, *VEGF*, and *STAT5B* are key nodes in the “Cardiovascular Disease, Lipid Metabolism, Molecular Transport” network and also interact with other DE genes.

Arnaud et al. [27], reported that *PTGS2* had capability to participate in heat stress-induced myocardial preconditioning *in vivo. HMOX1* also has an important role in cellular homeostasis because of its pro- and antioxidative function [28,29]. Bloomer et al. [30], observed that *HMOX1* was sensitive to temperature and its expression was increased immediately after heat stress. *PTGS2* can regulate *HMOX1* expression and this expression is up-regulated in cardiac fibroblasts when the broilers were under stress [31]. In addition, *HMOX1* can be regulated by c-fos induced growth factor (*FIGF*), a member of the PDGF/VEGF family,that can associate with mitogenic and morphogenic activity on fibroblast cells [32,33]. *PDGFB* can be affected by *PTGS2*, *HMOX1*, actin, alpha, cardiac muscle 1 (*ACTC1*), myosin, heavy chain 11, smooth muscle (*MYH11*), bone morphogenetic protein 3 (osteogenic) (*BMP3*). Signoroni et al. [34], demonstrated that *PTGS2* can modulate constitutive interaction of a PDGF receptor. *ACTC1* plays a crucial role in early muscle development and fetal development [35,36], and reduced *ACTC1* expression had an essential role in congenital heart disease and atrial septa defect [37,38]. *ACTC1* also is involved in “Cell-to-Cell Signaling and Interaction, Molecular Transport, Small Molecule Biochemistry” and “Lipid Metabolism, Molecular Transport, Small Molecule Biochemistry”. Many research studies [39,40] have indicated that gene *ACTC1* contributes to muscle development and meat quality. *MYH11* is a unique gene of smooth muscle cells. Landreh et al. [41], suggested that the cultured PDGF receptor α- positive peritubular cell (PTC) can increase *MYH11* (PTC-specific gene) expression. PDGF receptorα- positive PTC is a steroidogenic member of the stem Leydig cell (SLCs). BMP3 has been identified as playing a crucial role in both skeletal development and bone homeostasis [42].

In this study, we found that *PTGS2*, *FIGF*, *ACTC1*, *MYH11*, and *BMP3* were up-regulated under heat stress and their fold changed from 1.51 to 15.14 while the expression of *HMOX1* and *PDGFB* decreased during heat stress and their fold changes were −1.64 and −1.80, respectively. All these DE genes are in HS vs. CL or HS vs. TR. However, except for *HMOX1*, those DE genes have not been reported as relating to heat stress. This might be relatively few researchers have studied meat quality and growth under heat stress at the gene level. Thus, based on this study, we can infer that heat stress significantly influences broiler body temperature, meat quality, sperm quality, growth, and skeletal quality through up-regulation or down-regulation of unique genes in the hypothalamus. Furthermore, the changes of expression of muscle-related genes indicate that muscle development is inhibited to a certain degree in the short term after heat treatment.

Heat stress also can change the hormone level. Tyrosine hydroxylase (*TH*), the precursor of catecholamines (the sympathetic nervous system) (SNS) neurotransmitters, is a rate-limiting enzyme for catecholamine biosynthesis and biomarker enzyme for adrenaline expression [16,43]. In this study, *TH* was a key node and involved in “Cardiovascular Disease, Lipid Metabolism, Molecular Transport” and “Lipid Metabolism, Molecular Transport, Small Molecule Biochemistry” networks. It can catalyse tyrosine into dopa that can be transformed into catecholamine by the dopamine enzyme. *TH* can also regulate the receptor of dopa (*DRD2*) amd dopa could also be formed into noradrenaline and adrenaline through a special enzyme. Garcia et al. [44], reported that *TH* expression was decreased under chronic heat stress. However, in our acute heat-stress study, *TH* and *DRD2* were both significantly expressed with 14.91 and 1.63 FC, respectively. These genes are DE expressed in both HS vs. CL and HS vs. TR. Our current findings reveal that host-enhanced SNS activity leads to an up-regulated catecholamine metabolic pathway and increases noradrenaline and adrenaline to reinforce a body’s resistance to heat stress.

There are also still a number of interested DE genes in the three gene networks but for reasons of space not discussed here; they included *SDC1*, *EMP1*, *EDN3*, *DBI*, *INSIG1*, *HDL*, *CETP*, *PDK4* in “Cardiovascular Disease, Lipid Metabolism, Molecular Transport”, *GCH1*, *LMX1A*, *MOV10L1*, *GDPD5*, *JPH*, *AOAH* in “Cell-to-Cell Signaling and Interaction, Molecular Transport, Small Molecule Biochemistry”, *CUBN*, *MYH7*, *CROT*, *LPAP4*, *PLA1A* in “Lipid Metabolism, Molecular Transport, Small Molecule Biochemistry”. All these DE genes were related to meat quality, growth, and enzyme; for example, *MYH7* is related to drip loss and cooking loss [45,46]. More details are given in a supplemental file. Moreover, some of those DE genes have been identified as being associated with meat quality under heat stress by other researchers. For example, Yu et al. [47], reported that myogenic factor 6 (*MYF6*) and fatty acid binding protein 4, adipocyte (*FABP4*) were involved in meat quality under heat stress.

### Heat shock protein

During heat stress, a class of heat shock protein family could also be significantly changed. When a host encounters heat stress, the expression of highly-conserved heat-shock protein (HSP) will be either increased or decreased in various tissues and cells to protect the body from excessive damage [48]. HSP are ubiquitous in organisms ranging from from bacteria to mammals throughout the whole biosphere. They can help the body adapt to adverse environments by serving as molecular chaperone, antioxidant, synergistic immune, and anti-apoptotic [49]. HSP70 can also play a significant role in heat stress, but we didn’t find that it was DE in the current study. This might be because of the distribution of HSP70 in terms of different expression in diverse tissues or organs, perhaps especially enriching heart, liver, kidney, and spleen but not gathering in hypothalamus. In addition, HSP70 usually changes within eighteen hours, while after eighteen hours its expression may be back to normal [50]. In this study we found that the expression of *HSPCB* (HSP90) and *DNAJC13* (HSP40) underwent significant changes. *HSPCB* families can play a key role in DNA replication and transcription, protein folding and mutation, protein transport, proteolysis, cell signaling, and immune response [51,52]. *HSPCB* is also necessary in hormone receptor and tyrosine protein kinase [53,54]. *DNAJC13* can interact with HSP70 in renaturation of heat-denatured proteins [55] and *DNAJC13* can also bind directly to HSP70 by recruiting specific substrates to stimulate both its intrinsically weak ATPase activity and its interaction with polypeptide substrates [56,57]. *HSPCB* and *DNAJC13* are also DE in HS vs. CL and HS vs. TR. The expression of *DNAJC13* was up-regulated while the expression of *HSPCB* was down-regulated in the heat-stress process, indicating that heat stress can exert great harm to the body and the body can actively produce various mechanisms to resist heat stress.

## Conclusions

Transcriptome of the chicken hypothalamus could be altered extensively when chickens are exposed to heat stress. We performed global gene-expression analysis of hypothalamus from chickens subjected to heat stress, temperature recovery, and normal temperature. We used bird hypothalamus as material and used a microarray to detect the versatile transcript changes respond to broiler heat stress. In hypothalamus, the TR group and the CL group behaved similarly to the HS group. Various enzymes are changed following the level of hormone under heat stress. At the same time, a number of DE genes related to meat quality and growth were significantly DE. The expression of heat shock protein families also presented certain changes, especially in *DNAJC13* and *HSPCB*. The results of this study provides novel insight into the effects of heat stress on meat quality, growth, and essential enzyme for hormone pathway by using hypothalamus transcriptome analysis; it offers a platform for future investigations into genetic networks for studying broiler heat-stress response.

## Additional file

Additional file 1: Table S1. List of some differentially expressed (DE) genes in muscle, growth and enzyme.
