# Supplementary material for: Transcriptome responses to heat stress in hypothalamus of a meat-type chicken
Source: J Anim Sci Biotechnol. 2015 Feb 17;6(1):6. doi: 10.1186/s40104-015-0003-6 (PMC4359534; doi:10.1186/s40104-015-0003-6)
Supplement: Additional file 1: Table S1. — List of some differentially expressed (DE) genes in muscle, growth and enzyme. [file 40104_2015_3_MOESM1_ESM.docx]

## Table S1. List of some differentially expressed (DE) genes in muscle, growth and enzyme

| Gene symbol | Gene description | Genbank accession number | Fold change | GO term annotation |
| --- | --- | --- | --- | --- |
| DE genes related to meat quality | | | | |
| ACTC1 | actin, alpha, cardiac muscle 1 | NM_001079481 | 15.14 | GO:0030048 actin filament-based movement; GO:0030240 muscle thin filament assembly |
| CETP | cholesteryl ester transfer protein, plasma | NM_001034814 | 3.43 | GO:0042632 cholesterol homeostasis |
| CROT | similar to carnitine O-octanoyltransferase (LOC420533) | XM_418635 | 19.35 | GO:0015908 fatty acid transport |
| CUBN | cubilin (intrinsic factor-cobalamin receptor) | CR387507 | -1.61 | GO:0042953 lipoprotein transport |
| DBI | diazepam binding inhibitor (GABA receptor modulator, acyl-Coenzyme A binding protein) (DBI) | NM_204576 | 1.65 | GO:0008289 lipid binding |
| FABP7 | fatty acid binding protein 7 | NM_205308 | 1.88 | GO:0008289 lipid binding |
| GDPD5 | glycerophosphodiester phosphodiesterase domain containing 5 | N/A | 1.68 | GO:0006629 lipid metabolism;  GO:0007399 nervous system development |
| JPH1 | junctophilin 1 | CR353454 | 1.66 | GO:0007517 muscle development |
| LIPG | endothelial lipase | TC283514 | -1.55 | GO:0006629 lipid metabolism;  GO:0042632 cholesterol homeostasis; GO:0050746 regulation of lipoprotein |
| LOC430178 (MOV10L1) | similar to MOV10-like 1 | XR_027177 | 1.63 | GO:0007517 muscle development; GO:0008283 cell proliferation |
| MYH7 | myosin, heavy chain 7, cardiac muscle, beta | NM_001001302 | -2.73 | GO:0006941 striated muscle contraction |
| MYH11 | myosin, heavy chain 11, smooth muscle | NM_205274 | 3.27 | GO:0006939 smooth muscle contraction; GO:0030241 muscle thick filament assembly |
| PLA1A | Phospholipase A1 member A | TC283048 | -1.51 | GO:0006629 lipid metabolism |
| PTGS2 | Prostaglandin G/H synthase 2 Precursor | M64990 | 1.51 | GO:0006633 fatty acid biosynthesis; GO:0001516 prostaglandin biosynthesis |
| SDC1 | syndecan 1 | XM_419972 | -1.51 | GO:0055002 striated muscle cell development |
| UCP3 | uncoupling protein 3 | NM_204107 | -1.56 | GO:0006631 fatty acid metabolism |
| MYF6 | myogenic factor 6 (herculin) | NM_001030746 | -3.13 | GO:0007517 muscle development |
| VIT2 | vitellogenin 2 | NM_001031276 | 2.59 | GO:0006869 lipid transport |
| APOLD1 | apolipoprotein L domain containing 1 | XM_001233202 | -2.24 | GO:0006869 lipid transport;  GO:0042157 lipoprotein metabolism |
| P20K | quiescence-specific protein | NM_205422 | 3.21 | GO:0032496 response to lipopolysaccharide; GO:0055089 fatty acid homeostasis |
| RCJMB04_1d1 (INSIG1) | insulin induced gene 1 | NM_001030966 | -1.54 | GO:0006629 lipid metabolism;  GO:0008202 steroid metabolism;  GO:0008203 cholesterol metabolism |
| RCJMB04_9i11 | acyl-CoA synthetase bubblegum family member 2 | NM_001012846 | -1.52 | GO:0006629 lipid metabolism;  GO:0006631 fatty acid metabolism |
| AOAH | hypothetical LOC420737 | XM_418835 | 1.81 | GO:0006629 lipid metabolism;  GO:0008653 lipopolysaccharide metabolism |
| LOC776729 | similar to Zcwpw2 protein | XM_001236232 | 1.59 | GO:0007517 muscle development; GO:0008283 cell proliferation |
| DE genes related to growth | | | | |
| BMP4 | bone morphogenetic protein 4 | NM_205237 | -1.59 | GO:0008083 growth factor activity; GO:0001501 skeletal development |
| EMP1 | epithelial membrane protein 1 | BX930381 | -1.69 | GO:0016049 cell growth |
| EDN3 | Endothelin 3 | AB235921 | 1.69 | GO:0005179 hormone activity |
| LMX1A | LIM homeobox transcription factor 1, alpha | BM426154 | 1.64 | GO:0001558 regulation of cell growth |
| FIGF | c-fos induced growth factor | NM_204568 | 2.18 | GO:0008083 growth factor activity |
| SMAD7B | TGF-beta signal pathway antagonist Smad7 | NM_001159663 | 1.62 | GO:0007179 transforming growth factor beta receptor signaling pathway |
| BMP7 | bone morphogenetic protein 7 | XM_417496 | 1.58 | GO:0008083 growth factor activity |
| BMP3 | bone morphogenetic protein 3 | NM_001034819 | 1.71 | GO:0008083 growth factor activity |
| DE genes related to enzyme | | | | |
| DRD2 | dopamine receptor D2 | NM_001113290 | 1.63 | GO:0001670 dopamine D2 receptor activity; GO:0004871 signal transducer activity |
| GCH1 | GTP cyclohydrolase 1 (dopa-responsive dystonia) | NM_205223 | 1.95 | GO:0003824 catalytic activity;  GO:0042416 dopamine biosynthesis |
| GPR23 (LPAR4) | G protein-coupled receptor 23 | BX932026 | 1.52 | GO:0007186 G-protein coupled receptor protein signaling pathway |
| PDK4 | pyruvate dehydrogenase kinase, isozyme 4 | CR387492 | 2.25 | GO:0004673 protein histidine kinase activity; GO:0016772 transferase activity, transferring |
| TH | tyrosine hydroxylase | NM_204805 | 14.91 | GO:0004511 tyrosine 3-monooxygenase activity |
| HMOX1 | heme oxygenase (decycling) 1 | NM_205344 | -1.80 | GO:0002246 healing during inflammatory response; GO:0006788 heme oxidation |
